# Combined hepatocellular-cholangiocarcinoma: An analysis of clinicopathological characteristics after surgery

**DOI:** 10.1097/MD.0000000000017102

**Published:** 2019-09-20

**Authors:** Hao Zhang, Xiaojiong Yu, Jian Xu, Juan Li, Yao Zhou

**Affiliations:** a Department of Hepatobiliary Surgery; b Department of Pathology, The First Hospital Affiliated to University of Electronic Science and Technology of China & Sichuan Provincial People's Hospital, Chengdu, Sichuan, PR China.

**Keywords:** Chinese, hepatectomy, liver cancer, survival rate

## Abstract

Combined hepatocellular-cholangiocarcinoma (CHCC) is a rare type of primary liver cancer (PLC). The aim of this study was to investigate the disease characteristics in CHCC patients and compare them with those in hepatocellular carcinoma (HCC) or intrahepatic cholangiocarcinoma (ICC).

The perioperative and follow-up data of CHCC patients (n = 15), HCC patients (n = 577), and ICC patients (n = 61) were retrospectively analyzed, and the clinicopathological characteristics were compared among these 3 groups.

In the CHCC group, the serum level of AFP was significantly higher than that of the ICC group (*P* = .002), and the CA19-9 level was higher than that of the HCC group (*P* = .011). The positive rates of CK7 and CK19 expression were higher in CHCC group than in HCC group (both *P* < .001), while the positive rates of Glypican–3 and Hepatocyte expression were higher in CHCC group than in ICC group (both *P* < .001). Meanwhile, the CHCC patients were likely to have undergone more MJH/LT than the HCC patients (*P* = .037) and the ICC patients (*P* = .011). Macrovascular invasion and lymph node metastasis in the CHCC group were significantly higher but satellite lesions were similar, compared to the HCC group. Both the 1-year disease-free survival (DFS) and the 1-year overall survival (OS) for the CHCC patients were worse than those for the HCC patients. AFP ≥ 400 ng/ml, tumor size ≥5 cm, tumor number ≥2, macro- and microvascular invasion, distant metastasis and positive margin were risk factors for both DFS and OS for the PLC patients. Multivariate analysis also confirmed that ICC and lymph node metastasis were risk factors for DFS and MJH/LT was risk factor for OS.

CHCC patients appear to have intermediate clinical characteristics in comparison with the HCC and ICC patients, and the 1-year DFS and OS for the CHCC patients was worse than the HCC patients, but similar to the ICC patients.

## 1 Introduction

Combined hepatocellular-cholangiocarcinoma (combined HCC-CC; CHCC) is a rare type of primary liver cancer (PLC). It accounts for 1.0% to 4.7% of PLC, with the incidence varying among multiple studies.^[1]^ This type of carcinoma contains unequivocal intimately mixed components of both hepatocellular carcinoma (HCC) and intrahepatic cholangiocarcinoma (ICC).^[2,3]^ The first comprehensive description^[4]^ regarding CHCC was published in 1949 by Allen and Lisa. It was defined as the intimate intermingling of both HCC and ICC components and was classified into 3 types: type A (double cancer; HCC and ICC present at different sites within the same liver), type B (combined type; HCC and ICC present at adjacent sites and mingled with continued growth), and type C (mixed type; HCC and ICC components are combined within the same tumor). Then, the different definitions and classification methods for CHCC were issued in different research. The most common ones were the improved type of Goodman^[5]^ in 1985 and the Libbrecht type^[6]^ in 2006. The former updated the classification as

(1) collision tumors, a coincidental occurrence of both HCC and ICC in the same patient;
(2) transitional tumors including areas of intermediate differentiation and an identifiable transition between HCC and CC; and
(3) fibrolamellar tumors, which resemble the fibrolamellar variant of HCC but also contain mucin-producing pseudoglands.

On the basis of the improved type of Goodman, the latter (Libbrecht type) changed the third type to intermediate subtype (hepatocyte-cholangiocyte), CHCC that consist (almost) completely of such transitional areas and contain only very limited or no unequivocal hepatocellular and cholangiocellular components. The latest one was the World Health Organization (WHO) classification^[7]^ in 2010. According to the recent WHO classification, CHCC is divided into the classical type and subtypes with stem cell features. The former is similar to type C described by Allen and Lisa; and the latter is subdivided into the typical subtype, intermediate cell subtype, and cholangiolocellular subtype, according to the histopathology and immunophenotype.

An accurate preoperative diagnosis of CHCC is difficult,^[8]^ and most cases are confirmed by postoperative histopathology. As we know, patients with HCC are more likely to present with elevated AFP, increased expression of Glypican–3 and Hepatocyte, as well as more vascular invasion. While the clinical manifestations of ICC patients were mostly higher level of CA19-9, increased expression of CK7 and CK19, and early lymph node metastasis. But for CHCC, controversies exist regarding its clinicopathological characteristics and prognostic features. Some investigators have suggested that the biological features of CHCC resemble those of HCC rather than those of ICC.^[9]^ Meanwhile, another report shows that CHCC is genetically more similar to ICC than HCC.^[10]^ In this regard, there is currently no unified understanding. Therefore, we attempted to compare the clinicopathological characteristics and prognostic features of patients with CHCC to those with HCC or ICC who had undergone an operation during the same study period to obtain more accurate conclusions.

## 2 Materials and methods

### 2.1 Patients

Of the 705 patients who had been underwent a hepatectomy for PLC from January 2013 to December 2017 at Sichuan Academy of Medical Sciences & Sichuan Provincial People's Hospital, People's Republic of China, 17 were diagnosed with CHCC, including 15 who were classified as type C by Allen and Lisa,^[4]^ 1 type A, and 1 type B. But according to the latest definition of CHCC,^[7]^ we excluded the 2 patients classified as type A or type B. Therefore, only 15 patients were enrolled in this study. At the same time, the exclusion criteria for all patients were listed as below:

(1) The other types of PLC, including hepatoblastoma, angiosarcoma, or other specified liver carcinomas as well as those with hilar cholangiocarcinoma, were excluded;
(2) The patients with initial diagnosis and operative time earlier than study were excluded;
(3) The patients accompanied with other types of malignant tumors within 3 years were excluded.

After ruling out the patients according to the exclusion criteria, 15 patients, 577 HCC and 61 ICC patients were enrolled in this study. All intrahepatic ICC patients had the mass-forming macroscopic subtype. This study was approved by the institutional review board of our hospital.

### 2.2 Methods

The clinical information, including patient age at diagnosis, gender, alcohol consumption, viral hepatitis B and C status, serological data, background liver disease, and outcome of preoperative computed tomography (CT) or magnetic resonance imaging (MRI), was obtained from hospital records. Also, the Child-Pugh status was defined for each patient. The standard drink was defined as one 350 ml bottle of beer, one 150 ml glass of wine, or 50 ml of distilled spirits. The definition of alcohol consumption is a person drinks more than the standard drink a day for more than 3 years, and without recent alcohol withdrawals. Based on the number of liver segments lost by hepatectomy, the surgical method^[11]^ was categorized as minor hepatectomy (MNH), in which 1 or 2 segments containing the tumor were resected; major hepatectomy (MJH), in which more than 2 segments were resected, and liver transplantation (LT). Furthermore, LT was performed according to Fudan criteria.^[12]^ Routine dissection of lymph nodes was performed for ICC patients. These patients were staged according to the 8th edition American Joint Committee on Cancer criteria. The data of pathological reports, metastasis, and follow-up were recorded. Serum concentrations of α-fetoprotein (AFP) and carbohydrate antigen 19-9 (CA19-9) were measured for all patients at approximately 1 month after the treatment. Thereafter, all patients were regularly monitored for any intrahepatic recurrence or distant metastasis every 3 months in the first 2 years, with measurement of serum tumor markers, liver function tests, chest radiography, and CT or MRI scan.

### 2.3 Statistical analysis

Continuous variables are shown as the mean ± standard deviation, while categorical variables are shown as the frequency (n) with percentage (%). The least significant difference *t* test was used to compare continuous variables between two groups, and univariate analysis of variance was used to compare continuous variables among three groups. Categorical variable comparisons among 3 groups were performed by Pearson's χ^2^ test, and comparisons between two groups were performed by the partition χ^2^ method. Spearman correlation analysis was used to compare the correlation among variables. Univariate and multivariate Cox regression analyses were used to calculate the hazard ratios (HRs) and 95% confidence intervals (CIs) for disease-free survival (DFS) and overall survival (OS), respectively.^[14]^ The Kaplan–Meier method was used to estimate the cumulative incidences of events, and differences in these incidences were evaluated using the log–rank test. Two-sided *P* < .05 was regarded as having statistical significance. The SPSS 22.0 Statistical Package (IBM SPSS STATISTICS) was used for all analyses.

## 3 Results

The baseline characteristics of the CHCC, HCC, and ICC patients are provided in Table 1. There were 12 men (80.0%) in the CHCC group, and the mean age was 55.07 ± 12.16 years old. Compared to the HCC and ICC groups, respectively, no significant difference was seen in terms of the gender distribution or mean age. Eight patients (53.3%) with CHCC had hepatitis B virus (HBV) or hepatitis C virus (HCV) infection, which were lower than those with HCC (*P* = .016), but higher than ICC (*P* < .001) groups. The serum level of AFP in the CHCC group was significantly higher than that of the ICC group (*P* = .002), but similar to that of the HCC group (*P* = .645). Also, the CA19-9 level in the CHCC group was higher than that of the HCC group (*P* = .011), but similar to that of the ICC group (*P* = .306). Furthermore, 4 patients (26.7%) in the CHCC group had elevated CA19-9 and AFP levels, which was higher than 12 patients (2.1%) in the HCC group (*P* < .001) or 0 patient in the ICC group (*P* < .001). Twelve Child-Pugh C patients were enrolled in this study which was suffering HCC. No significant difference was seen in terms of the Child-Pugh status or alcohol consumption among the 3 groups (Table 1).

**Table 1:**
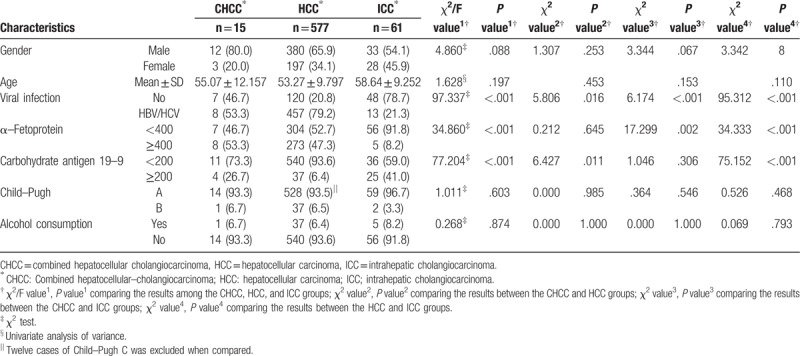
Baseline characteristics of CHCC, HCC, and ICC patients.

The CHCC patients were more likely to have undergone an MJH/LT than the HCC patients (*P* = .037) and the ICC patients (*P* = .011). In CHCC group, the multiple nodules tumor was more regular than in ICC group (*P* = .047). Both microvascular invasion and macrovascular invasion were more regular in the CHCC group than in ICC group (both *P* < .05). But compared to HCC group, only macrovascular invasion was more frequently (P < .05). Positive margins, satellite lesions, and cirrhosis were not significantly different among all three groups (all *P* > .05). Lymph node metastasis was more frequent in the CHCC group than in HCC group (*P* < .001) (Table 2).

**Table 2:**
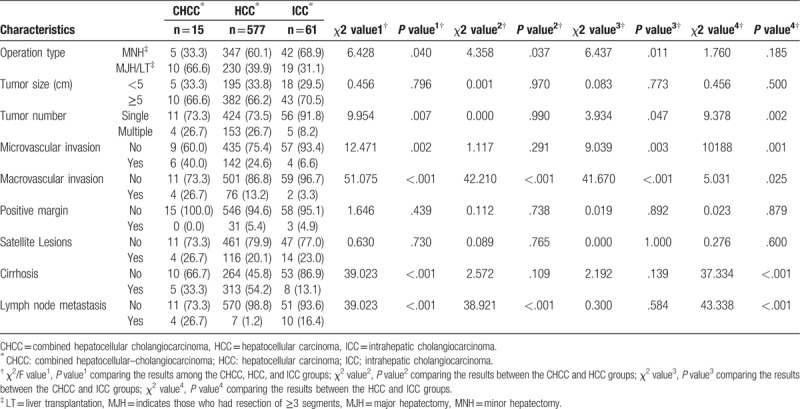
Surgical methods and pathological characteristics of CHCC, HCC, and ICC patients.

The percentage of CD34- and Ki-67-positive patients^[13]^ in the CHCC group was similar to those in the 2 other groups. Both the distributions of CK7- and CK19-positive patients in the CHCC group were similar to those in the ICC group (both *P* > .05), but higher than those in the HCC group (both *P* < .05). On the contrary, the ratios of glypcian-3 and hepatocyte-positive patients in the CHCC group were similar to those in the HCC group (both *P* > .05), but higher than those in the ICC group (both *P* < .05) (Table 3).

**Table 3:**
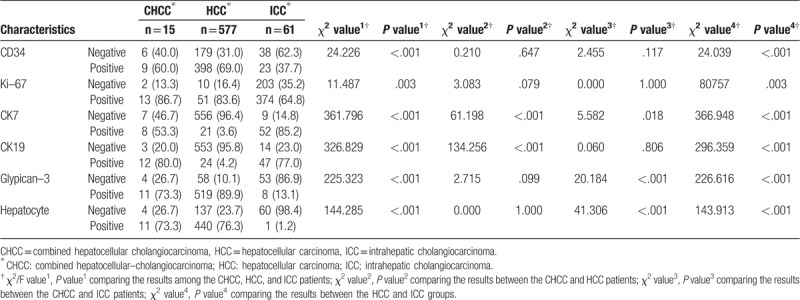
Immunohistochemical characteristics of CHCC, HCC, and ICC patients.

**Figure 1:**
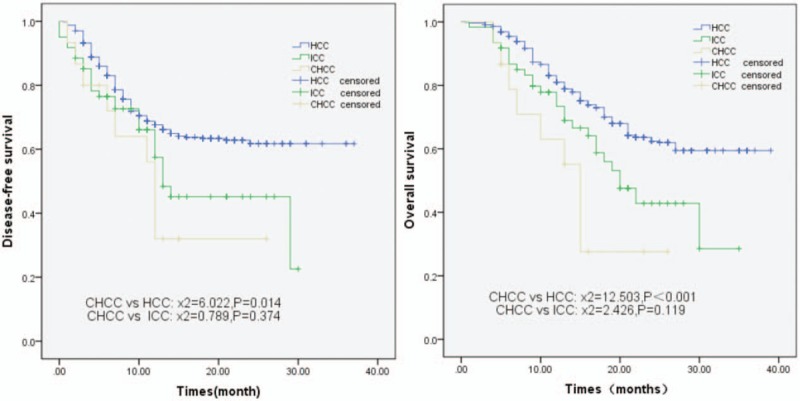
Disease-free survival rate (A) and overall survival rate (B) of combined hepatocellular cholangiocarcinoma, hepatocellular carcinoma, and intrahepatic cholangiocarcinoma patients.

Twelve of the 15 CHCC patients (80.0%) had recurrence during the follow-up period (from 3 to 35 months), and 10 of these patients died due to the recurrence. The median DFS was 12.0 months (95% CI: 7.2–16.8 months), and the median OS was 15.0 months (95% CI: 10.5–19.5 months). The 1-year DFS rates of the HCC, ICC, and CHCC patients were 69.8%, 58.5%, and 36.1%, respectively, indicating that the DFS rates were similar for the CHCC and ICC groups (*P* = .374), but lower than that for the HCC group (*P* = .014) (Fig. 1A). The 1-year OS rates of the HCC, ICC, and CHCC patients were 81.3%, 73.8%, and 59.6%, respectively, which showed the rate of the CHCC group was similar to ICC group (*P* = .119), but significantly lower than HCC group (*P *< .001) (Fig. 1B).

Spearman correlation analysis showed the viral infection was related to tumor number (ρ* *=* *−0.203, *P *< .001) and satellite lesions (ρ* *=* *−0.129, *P* = .001), but not to the tumor size, lymph node metastasis, major and microvascular invasion. At the same time, there were no link between alcohol consumption and tumor number, lymph node metastasis, major and microvascular invasion or liver cirrhosis. But it was related to tumor size and satellite lesions (Table 4). Between the patients with or without alcohol consumption, there were statistic difference in DFS (*P* = .026) or OS (*P* = .027).

**Table 4:**
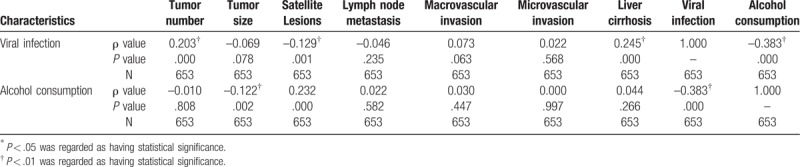
Correlation analysis by Spearman correlation.

According to Cox regression analysis for DFS, the univariate HR was estimated to be 0.442 (95% CI: 0.242–0.810, *P* = .008) for the HCC patients and 0.714 (95% CI: 0.365–1.397, *P* = .325) for the ICC patients, compared to the CHCC patients. However, in multivariate Cox regression, ICC patients increased the HR by 4-fold (95% CI: 1.652–9.619, *P* = .002) compared to CHCC patients. Gender, alcohol consumption, CA19-9 level, satellite lesions, operation type and ki-67 level did not influence the HR. But AFP level ≥400 ng/ml, tumor number ≥2, tumor size ≥5 cm, lymph node metastasis, distant metastasis, major and microvascular invasion and positive margin increased the HR by different fold respectively, according to multivariate analysis. (Table 5, Table 6).

**Table 5:**
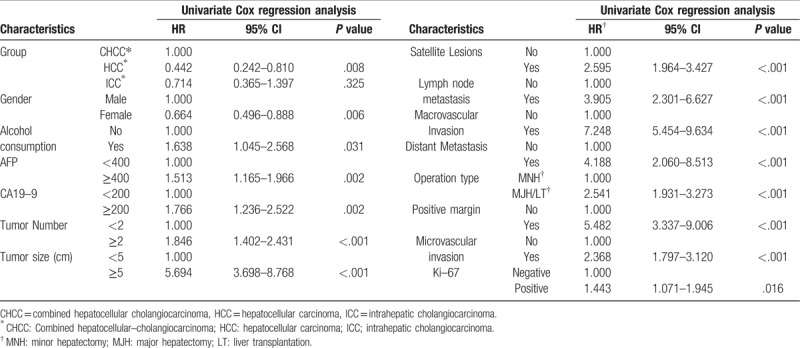
Disease–free survival analysis by Univariate Cox regression.

**Table 6:**
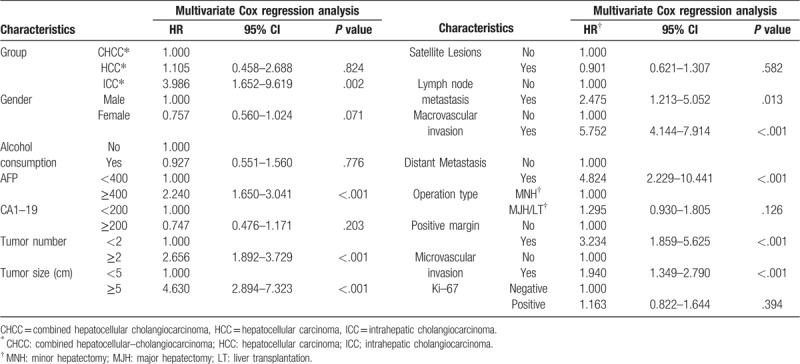
Disease–free survival analysis by Multivariate Cox regression.

By OS analysis, the univariate HR was estimated to be 0.325 (95% CI: 0.178–0.596, *P* < .001) for the HCC patients and 0.578 (95% CI: 0.295–1.131, *P* = .109) for the ICC patients, compared to the CHCC patients. Also, Gender, alcohol consumption, CA19–9 level, satellite lesions and ki-67 level did not influence the HR according to multivariate Cox regression analysis. Meanwhile lymph node metastasis did not increase the HR neither. Different from the multivariate analysis for DFS, MJH/LT increased the HR by 1.4-fold (95% CI: 1.015–1.967, *P* = .041). Furthermore, the AFP level ≥400 ng/ml, tumor number ≥2, tumor size ≥5 cm, distant metastasis, major and microvascular invasion and positive margin influenced the HR either. (Table 7, Table 8).

**Table 7:**
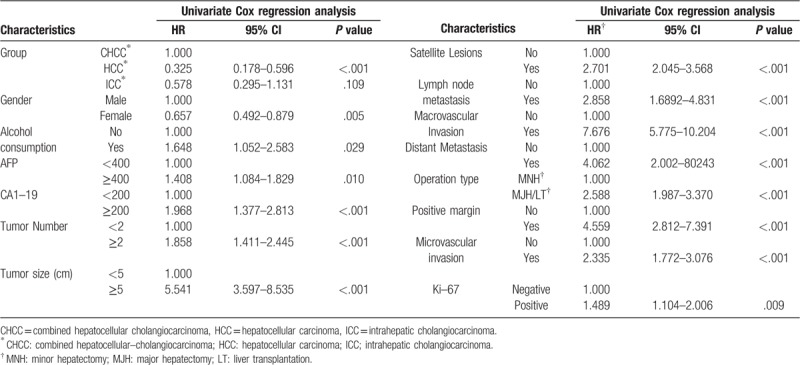
Overall survival analysis by Univariate Cox regression.

**Table 8:**
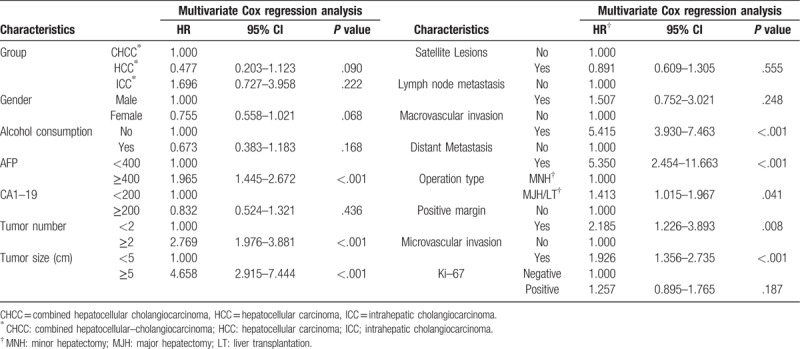
Overall survival analysis by Multivariate Cox regression.

## 4 Discussion

The etiology of CHCC is not clear. Most of the literature^[2]^ considers the source of the mixed type to be liver cancer stem cells. In addition, Ogasawara et al^[14]^ have confirmed in their research on mice that CHCC may originate from epithelial cell adhesion molecule-positive human hepatic neoplastic cells and that these cells may affect the malignant behavior and tumor progression. Moreover, Zhou et al^[15]^ have discovered that HBV infection and heavy alcohol consumption may play a role in the development of CHCC in Chinese patients.

Whether the clinicopathological characteristics of CHCC are similar to those of HCC or ICC is inconsistent in different studies. Among groups, the age at diagnosis and the gender ratio are similar in some reports.^[16,17]^ The rates of viral hepatitis infection are similar as well.^[17]^ However, Lee et al^[18]^ have found that HCC and ICC patients are younger than CHCC patients and that these diseases predominantly affect men. In our study, the gender at diagnosis were similar among groups, but men accounted for the majority in the CHCC and HCC groups. Moreover, the proportion of HBV and/or HCV infection in CHCC was lower than that in HCC, but higher than that in ICC. The serum AFP level was more elevated in the CHCC patients than in the ICC patients, and the serum CA19–9 level was higher than that in the HCC patients. Furthermore, the immunohistochemical characteristics of the CHCC patients had intermediate features of HCC and ICC. Both the ICC-specific markers (CK7 and CK19) and the HCC-specific markers (glypcian-3 and hepatocytes) were elevated in the CHCC patients. These results were consistent with the findings of Lee et al.^[19]^

Before surgery, CHCC can be misdiagnosed as HCC or ICC until the pathological outcome is obtained.^[20]^ An increase of both the serum AFP and CA19-9 levels may be helpful in raising a suspicion of CHCC,^[6]^ and three patients in our study were diagnosed in this way and confirmed by pathological results. Some studies^[20,21]^ have shown the preoperative diagnosis of CHCC based on imaging features. Ijichi et al^[22]^ also have suggested that fluorodeoxyglucose-positron emission tomography may be a useful diagnostic tool for the preoperative evaluation of the aggressiveness of PLCs, such as CHCC. But in the absence of classic imaging features, a radiologist cannot make an accurate diagnosis, and a biopsy should be considered.

When comparing the surgical features of CHCC to HCC and ICC in our study, a MJH was performed more frequently in CHCC patients than in HCC patients and ICC patients, including 2 patients with a LT. Magistri et al^[23]^ preferred to choose a MJH instead of a LT for CHCC, when acceptable outcomes could be obtained. Also, Garancini et al^[11]^ identified 465 patients with CHCC, 52,825 patients with HCC, and 7181 patients with ICC and reported that a LT in the CHCC patients showed inferior survival in comparison with HCC patients and that a MJH may be considered the best therapeutic approach. From the above results, we considered that a MJH should be the preferred treatment for CHCC patients.

Vascular invasion, intrahepatic satellite metastases, and lymph node metastasis of CHCC would be reasons for a more advanced tumor status, and these findings collectively suggested a poor outcome after surgery.^[17,24,25]^ In our study, we found that macrovascular invasion and lymph node metastases in the CHCC group were significantly higher but satellite lesions were similar, compared to the HCC group. According to these, we hypothesized that macrovascular invasion and lymph node metastasis may be the major factors influencing the prognosis because, in our study, we obtained lower 1-year DFS rates and poorer 1-year OS rate for the CHCC group, compared with the HCC group. Also, we could obtain that macro- and microvascular invasion in the CHCC group were significantly higher but satellite lesions and lymph node metastasis were similar, compared to the ICC group. Meanwhile, vascular invasion is one of the clinical features of HCC, while lymph node metastasis is more common in ICC, which also indicates that CHCC has the intermediate clinical characteristics in comparison with the HCC and ICC patients.

## 5 Conclusions

CHCC contains 2 types of carcinoma cells simultaneously and shows some intermediate characteristics between HCC and ICC. Elevated AFP and/or CA19-9 levels could be observed, and a higher serum level of both should indicate a suspicious diagnosis of CHCC. More macrovascular invasion and lymph node metastasis were observed in the CHCC patients than in HCC patients, and most cases tested positive for CK-7, CK-19, glypcian-3, and hepatocytes by immunohistochemical analyses. The 1-year DFS and OS rates were worse for the CHCC patients, compared to the HCC patients. Therefore, a MJH should be considered as the preferred treatment for this type of malignant tumor.

## Author contributions

**Conceptualization:** Hao Zhang, Xiaojiong Yu, Jian Xu.

**Data curation:** Hao Zhang, Yao Zhou.

**Formal analysis:** Juan Li.

**Methodology:** Hao Zhang, Jian Xu.

**Project administration:** Jian Xu.

**Supervision:** Xiaojiong Yu, Jian Xu.

**Writing – original draft:** Hao Zhang, Yao Zhou.

**Writing – review & editing:** Hao Zhang.

Hao Zhang orcid: 0000-0002-1850-0770.
